# The causal effect of juvenile idiopathic arthritis on IgA nephropathy: A Mendelian randomization study

**DOI:** 10.1097/MD.0000000000048981

**Published:** 2026-06-26

**Authors:** Ling Kong, Hua Zou, WenYuan Xu, XiaoHui Xu

**Affiliations:** a National Clinical Research Center of Kidney Diseases, Jinling Hospital, Nanjing University School of Medicine, Nanjing China.

**Keywords:** IgA nephropathy, juvenile idiopathic arthritis, Mendelian randomization

## Abstract

IgA nephropathy (IgAN) has been documented in patients with various comorbidities. Previous observational studies showed an association between juvenile idiopathic arthritis (JIA) and IgAN. The aim of this study was to determine whether there is a causal effect of JIA on the risk of IgAN using a 2-sample Mendelian randomization (MR) approach. A 2-sample MR analysis was conducted to elucidate the potential causal relationship between JIA and IgAN. Summary-level data from genome-wide association studies in the European population were utilized, including IgAN (5556 cases and 21,178 controls) and JIA (3305 cases and 9196 controls). The inverse variance weighted method showed significant evidence of a positive causal relationship between genetically predicted JIA and IgAN. For each standard deviation increase in genetically predicted JIA, the risk of IgAN was found to be increased by 22% (odds ratio [OR] = 1.22, 95% CI: 1.10–1.29, *P* = 3.03 × 10^−4^). Similar associations were observed with the weighted median (OR: 1.16, 95% CI: 1.05–1.29) and weighted mode methods (OR: 1.16 95% CI: 1.02–1.33), but not with the simple median (OR: 1.22, 95% CI: 0.99–1.50) and MR Egger method (OR: 1.21, 95% CI: 0.93–1.57). Reverse MR analysis found no inverse causal relationship between these 2 diseases. This study provides genetic evidence supporting a causal link between JIA and an increased risk of IgAN. In JIA patients, periodic evaluation of kidney function and proteinuria is warranted.

## 1. Introduction

IgA nephropathy is the most common primary glomerulonephritis worldwide.^[1,2]^ IgA nephropathy (IgAN) is defined as a mesangial proliferative glomerulonephritis characterized by diffuse mesangial deposition of IgA. Data in our center show that slow progression to end-stage kidney disease occurs in 30% of patients within 20 to 25 years of presentation.^[3]^ IgAN has been documented in patients with various comorbidities, including gastrointestinal and liver disorders (chronic liver disease, Crohn’s disease, ulcerative colitis, celiac disease), other autoimmune disorders (rheumatoid arthritis, ankylosing spondylitis), respiratory tract diseases, viral infections, and neoplasia, etc.^[4]^ Although IgAN in those cases is often referred to as being secondary to the underlying systemic disorder, there is no consistent definition of secondary IgAN in the literature. In some conditions, IgAN may be pathogenetically related to the underlying condition, while in others the association may be coincidental.

Juvenile idiopathic arthritis (JIA) is a chronic idiopathic inflammatory disorder primarily involving joints, lasting for >6 weeks and with onset before the age of 16 years. It is referred to as “adult-onset Still’s disease” (AOSD) when it begins in patients ≥16 years of age. Despite being the most common chronic inflammatory rheumatic condition in childhood, the prevalence of JIA remains relatively low, with an estimated incidence of approximately 11.7 cases per 1,00,000 population per annum.^[5]^ Studies have demonstrated that children with JIA exhibit reduced estimated glomerular filtration rate (eGFR), increased urinary albumin excretion, and higher renal resistive index values compared to controls.^[6]^ The co-occurrence of JIA (or AOSD) with IgAN has been reported previously.^[7-11]^ However, it remains unclear whether JIA is a contributing factor to the development of secondary IgAN.

Mendelian randomization (MR) analysis is an epidemiological design that can strengthen causal inference by using genetic variants as instrumental variables (IVs) for an exposure.^[12-14]^ MR analysis utilizes genetic variants identified through genome-wide association studies (GWAS), which are randomly allocated at conception as IVs to investigate whether a lifetime exposure is causally associated with an outcome. An MR analysis showed that inflammatory bowel disease, ulcerative colitis, and Crohn’s disease were causally associated with the risk of IgAN.^[15]^ Recently, the results from a large-scale GWAS for IgAN and JIA were reported.^[16-18]^ The availability of these data allows the performance of further MR analyses. In the present study, therefore, we conducted 2-sample MR analyses to investigate the potential causal relationship between JIA and IgAN.

## 2. Materials and methods

### 2.1. Study design and data sources

A 2-sample MR analysis and a sensitivity analysis were performed to investigate whether genetically predicted JIA is causally associated with IgA nephropathy. The reverse association between IgAN and JIA was also investigated by the reverse MR analysis. The brief information of GWAS used in this study was summarized in Table 1. The GWAS summary statistics data for IgAN were derived from the latest study published by Kiryluk,^[16]^ including 11 European cohorts that include 5556 kidney biopsy-diagnosed IgAN cases and 21,178 controls. All the participants were of European ancestry. Details on the process of genotyping and quality control were described by Kiryluk et al.^[16]^ GWAS summary statistics data of JIA were retrieved from the GWAS catalog with catalog ID GCST90010715.^[17]^ Details on the process of genotyping and quality control were described by López-Isac et al.^[17]^ All the participants were of European ancestry (n = 3305 JIA patients and n = 9196 control subjects). As the effect allele frequency is not provided in the IgAN data set, the effect allele frequencies were imputed from the 1000 Genomes Project phase 3. None of the 2 GWAS summary datasets were adjusted for any covariables. There were no evident overlapping individuals among the 3 GWAS studies.

**Table 1:** Overview of genome-wide association studies used in this study.

GWAS dats set	Year	Exposures or outcomes	Cases	Controls	Catalog ID
López-Isac et al^[17]^	2020	Juvenile idiopathic arthritis	3305	9196	GCST90010715 (Primary)
Kiryluk et al^[16]^	2023	IgA nephropathy	5556	21,178	NA

GWAS = genome-wide association studies, IgAN = IgA nephropathy.

### 2.2. Selection of instrumental variables (IVs)

Genetic IVs for JIA data set GCST90010715 were selected according to the following criteria: SNPs were at the genome-wide significance level (*P* < 5 × 10^−8^). The SNPs in high linkage disequilibrium (*r*^2^ > 0.01, clump windows < 10,000 kb) were excluded based on the 1000 Genomes European reference panel. Palindromic IVs were excluded after data harmonization, as palindromic SNPs have intermediate allele frequencies. Additionally, an *F*-statistic threshold exceeding 10 was used to exclude genetic variations as potential IVs. In the reverse MR analysis, there were also only a few SNPs associated with IgAN at the level of *P* < 5 × 10^−8^, the genetic instruments were also set as *P* < 1 × 10^−6^. Other criteria were the same as the data set GCST90010715.

### 2.3. Statistical analyses

We primarily utilized the multiplicative random-effects inverse variance weighted (IVW) method to evaluate the impact of exposure to JIA on IgAN. The IVW method was considered the most efficacious approach when employing valid genetic IVs, particularly in the absence of pleiotropic effects associated with genetic IVs.^[13,14]^ The MR-Egger method was employed with Cochran’s *Q* statistic to evaluate heterogeneity. The IVW with random effects method was used when heterogeneity was statistically significant (*P* < .05). Otherwise, the IVW fixed-effects method was applied.^[14]^ Four supplementary analyses were used to confirm the results, including MR-PRESSO, weighted mode, weighted median, simple mode, and MR-Egger regression. A “leave-one-out” analysis was conducted to assess the impact of individual SNPs on the causal relationship between exposure and outcome. The MR-Egger intercept and MR-PRESSO tests were utilized to identify potential pleiotropy in the IVs.^[19]^ If horizontal pleiotropy is detected (Global test, *P* < .05) or outlier IVs are detected by the MR-PRESSO method, the outlier-corrected estimated causal effect would also be reported as a supplementary MR result.

All analyses were conducted using R (version 4.2.3) and the TwoSampleMR package (version 0.5.11). Associations with *P*-values < .05 were deemed statistically significant evidence of a causal association. All the GWAS summary-level data in this study were deidentified and publicly available. This study does not constitute human subjects research and therefore did not require institutional review board approval.

## 3. Results

### 3.1. Selection of valid genetic instrumental variables for JIA

A total of 21 SNPs related to JIA were selected as candidate IVs for MR analysis. One SNP (rs497523) was excluded for not appearing in GWAS data of IgAN, and one SNP (rs4766578) for being palindromic with intermediate allele frequencies. Finally, 19 SNPs were selected as genetic IVs for JIA. The *F*-statistics for every instrument exposure association were much >10 in this study, with average *F* values of 118.2. Thus, the SNP IVs are unlikely to be influenced by instrumental bias and are in accordance with the first hypothesis of MR analysis. The details including *P*-values, β coefficients, standard errors, and effect allele for the association between SNPs and JIA of the selected IVs are provided in Table S1, Supplemental Digital Content.

### 3.2. Genetically predicted JIA is associated with an increased risk of IgAN

**Figure 1.**
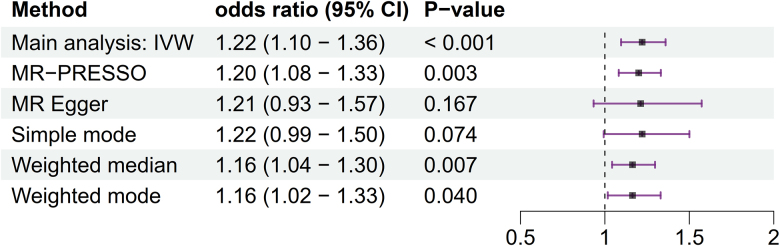
The causal association of genetically predicted juvenile idiopathic arthritis (JIA) on IgA nephropathy (IgAN). CI = confidence interval, IgAN = IgA nephropathy, IVW = inverse variance weighted (multiplicative random effects), JIA = juvenile idiopathic arthritis.

**Figure 2.**
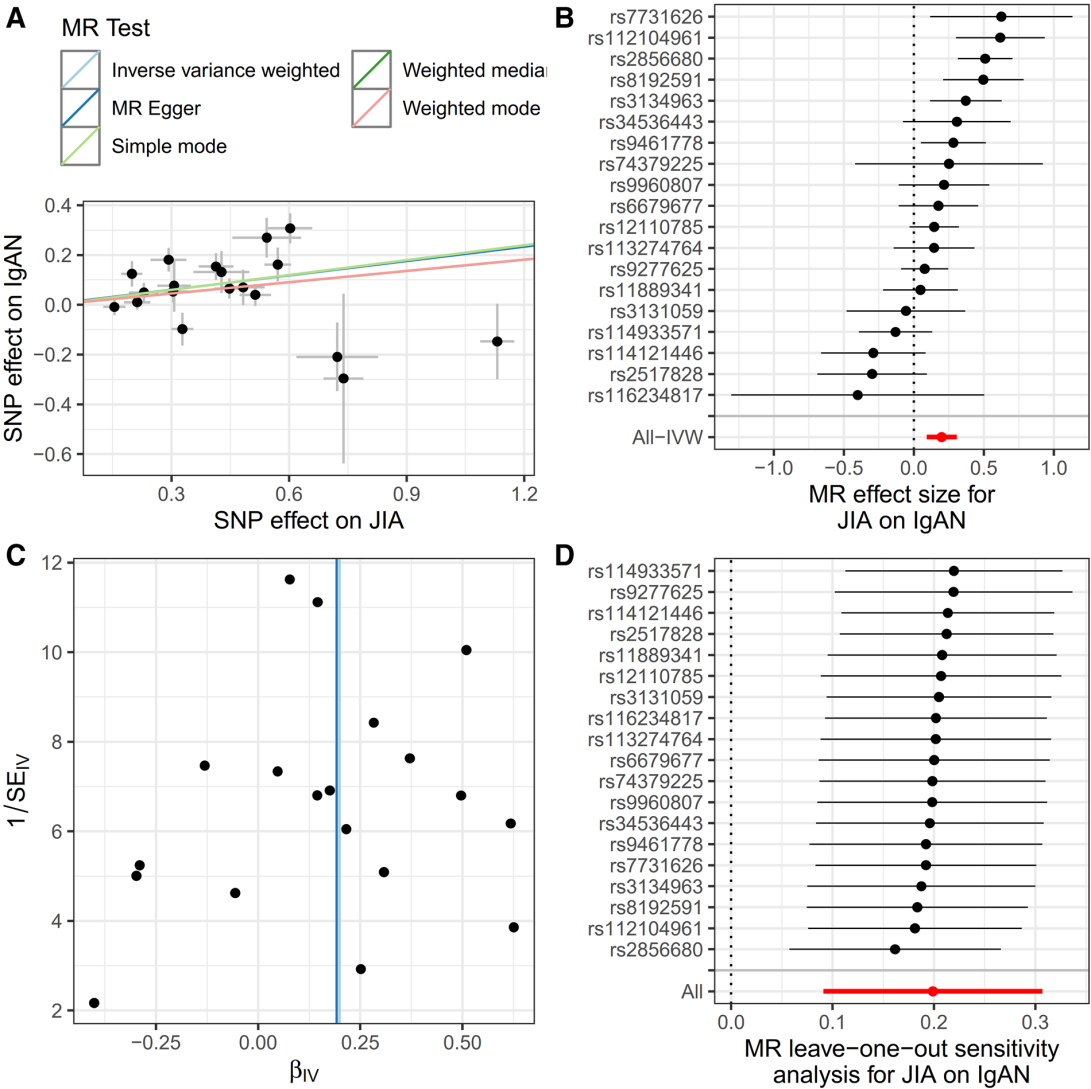
Scatter plot and sensitivity analysis of causal effects of juvenile idiopathic arthritis (JIA) on IgA nephropathy (IgAN). Panel A shows the scatter plots of SNP effects. The slope of each line corresponded to the estimated MR effect per method. The data are expressed as raw β values with a 95% confidence interval. Panel B is a forest plot illustrating the MR effects of individual SNPs. Panel C shows the funnel plot of the SNPs; panel D shows the leave-one-out sensitivity analysis. IgAN = IgA nephropathy, IVW = inverse variance weighted (multiplicative random effects), JIA = juvenile idiopathic arthritis.

Significant evidence was found supporting a positive causal relationship between genetically predicted JIA and IgAN, as illustrated in Figures 1 and 2A, B. In the primary MR analysis, the random-effects IVW was selected as the main result due to statistical heterogeneity identified by Cochran’s *Q* test (*P* < .001). For each standard deviation increase in genetically predicted JIA, the risk of developing IgAN increased by 22% (IVW odds ratio [OR] = 1.22, 95% CI: 1.10–1.29, *P* = 3.03 × 10^−4^). In the supplementary MR analysis, consistent associations were noted using the weighted median (OR: 1.16, 95% CI: 1.05–1.29) and weighted mode methods (OR: 1.16 95% CI: 1.02–1.33). However, these associations were not observed with the simple median method (OR: 1.22, 95% CI: 0.99–1.50) or the MR-Egger method (OR: 1.21, 95% CI: 0.93–1.57).

### 3.3. Sensitivity analysis

As previously noted, significant heterogeneity was detected in the MR analysis by Cochran’s *Q* test, and the funnel plot revealed a slightly asymmetrical distribution of individual SNP effects (Fig. 2C). No evidence of horizontal pleiotropy was found in the MR-Egger intercept analysis (MR-Egger intercept: 0.0027 ± 0.049, *P* = .95). The MR-PRESSO method identified 2 outlier SNPs (rs114933571 and rs2856680), yet their exclusion did not significantly alter the outcomes (OR: 1.18, 95% CI: 1.06–1.75). The causal relationship between genetically predicted JIA and IgAN, based on the remaining SNPs, remained consistent with the primary MR findings even after sequentially removing each SNP one by one, indicating the stability and robustness of this MR study (Fig. 2D).

### 3.4. Reverse MR analysis

**Figure 3.**
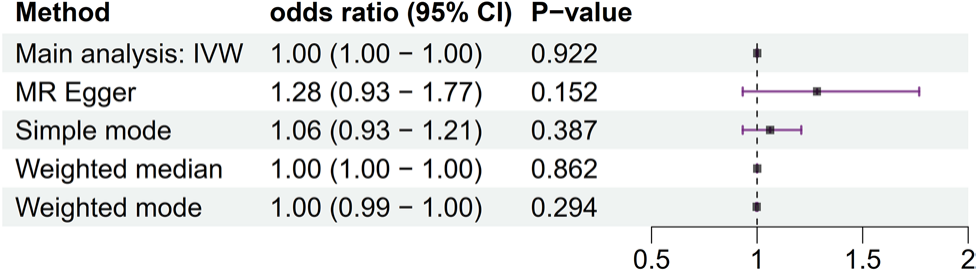
The results of reverse Mendelian randomization analysis to investigate the potential causal effect of IgA nephropathy (IgAN) on juvenile idiopathic arthritis (JIA). There was no horizontal pleiotropy and no outlier detected by the MR-PRESSO method in the reverse MR studies, thus the result of MR-PRESSO is not available. CI = confidence interval, IgAN = IgA nephropathy, IVW = inverse variance weighted (multiplicative random effects), JIA = juvenile idiopathic arthritis, MR = Mendelian randomization.

We conducted reverse MR analysis to explore the potential causal effect of IgAN on JIA. A total of 14 and 10 SNPs were screened and identified as IVs for the primary and validation data sets of JIA (Tables S2 and S3, Supplemental Digital Content). The *F*-statistics of all these SNPs are above ten with an overall *F*-statistic of 43.12. Nevertheless, none of the MR methods detected a significant reverse causal effect in either the primary or validation datasets for JIA (Fig. 3).

## 4. Discussion

In this study, we conducted 2-sample MR analyses to explore the causal association between JIA and IgAN, utilizing large-sample, cohort-based GWAS data. Our results indicate that a genetic predisposition to JIA was significantly associated with an elevated risk of IgAN. The positive causal effect of JIA on IgAN was further validated by sensitivity analysis. Although the associations in the simple median and MR-Egger methods did not reach statistical significance, likely due to lower statistical power compared to the IVW method, the direction of the effect estimates remained consistent (OR > 1), supporting the robustness of the primary IVW findings. Nonetheless, there was no compelling evidence supporting an inverse causal relationship between these 2 conditions. To the best of our knowledge, this is the first study to explore the potential bidirectional causal relationship between JIA and IgAN using the MR methodology. This study underscores the importance of interdisciplinary strategies in the management of JIA patients, particularly considering the increased risk of developing IgAN. Our findings suggest that screening for kidney involvement in JIA patients could be strategically informed, advocating for a vigilant approach towards monitoring renal function and proteinuria. Such proactive surveillance could pave the way for earlier interventions, potentially arresting the progression of IgAN in susceptible individuals.

IgAN has been reported to be associated with a diverse array of conditions including chronic liver disease, celiac disease, dermatitis herpetiformis, seronegative arthritis, small cell carcinoma, lymphoma, disseminated tuberculosis, bronchiolitis obliterans, and various autoimmune disorders.^[4]^ Some of these associations are recognized as forms of secondary IgAN, while others are based on anecdotal evidence. It is crucial to approach these latter observations with caution, as such associations might be coincidental rather than indicative of a direct causal link. JIA is a type of autoimmune disease. Given the relative rarity of both JIA and IgAN, it is not feasible to collect large samples in longitudinal studies to sufficiently study the causal relationship between JIA and IgAN. Fortunately, MR emerges as a valuable tool for identifying causal effects of exposure on outcomes.^[13]^ By leveraging genetic variations as IVs, MR operates under the assumption that these variations are distributed randomly and independently during meiosis, circumventing confounders and reverse causality issues. A previous MR study showed that inflammatory bowel disease, including ulcerative colitis and Crohn’s disease, had significant positive causal effects on IgAN risk.^[15]^

The relationship between JIA and IgAN, as revealed through MR analysis, provides novel insights into the potential etiological links between these conditions. However, the precise biological connection between JIA and IgAN remains undefined. IgAN is an autoimmune disease characterized by a dysregulated mucosal-type IgA immune response. Past studies suggest that disturbances in mucosal immunity and abnormal IgA responses are crucial in IgAN development.^[20,21]^ Interestingly, cross-sectional analysis revealed that JIA patients aged 10 to 17 exhibited elevated serum IgA levels compared to their age-matched counterparts.^[22]^ Additionally, the involvement of the complement system, indicated by C3 deposition along with IgA in most IgAN cases, points to a shared pathway of immune complex-mediated inflammation. This intersection suggests a wider scope of immune dysregulation, potentially linking JIA to renal issues like IgAN. Our findings, therefore, underscore the necessity for further research into the specific genetic factors involved and their roles in the intersecting mechanisms of these diseases.

Several limitations should be emphasized in this study. Primarily, the use of summary-level GWAS data from mainly European populations could restrict the applicability of our findings to different ethnic groups. The genetic variations that influence the risk of JIA and IgAN may vary among populations, highlighting the need for additional studies across a wider range of ethnic groups to confirm and enrich our results. Furthermore, MR analysis presumes the nonexistence of pleiotropy, meaning the instrumental genetic variants are assumed to influence the outcome exclusively through the exposure. Despite conducting sensitivity analyses, such as MR-PRESSO, to lessen the potential impact of pleiotropy, the existence of unmeasured pleiotropic effects remains a concern that could skew our conclusions. Future research, utilizing advanced methodologies and larger, more ethnically diverse cohorts, is imperative to further elucidate the genetic connections between JIA and IgAN. Regarding the terminology, AOSD and JIA describe the same clinical condition, differing only by the age at diagnosis. AOSD refers to individuals diagnosed after the age of 16, while JIA pertains to those diagnosed at or before the age of 16. It would be interesting to conduct MR analysis between IgAN and AOSD to validate the results of this study. However, AOSD is a rare disease with no published GWAS data available. Consequently, the MR analysis between IgAN and AOSD could not be performed in this study.

## 5. Conclusion

In summary, our MR study provides new evidence for a positive causal relationship between JIA and IgAN through a 2-sample MR approach. These findings pave the way for further research into the genetic and biological mechanisms underlying this association. Furthermore, they underscore the importance of proactive clinical surveillance for early indicators of IgAN in patients with JIA. Advancing our understanding of the genetic landscape of these conditions is essential for the development of precise therapeutic strategies aimed at preventing the progression of IgAN in individuals diagnosed with JIA. We recommend that rheumatologists and pediatricians perform routine screening for kidney involvement (e.g., urinalysis for hematuria/proteinuria) in children with JIA. This can lead to earlier diagnosis and better long-term renal survival.

## Author contributions

**Conceptualization:** XiaoHui Xu.

**Data curation:** Ling Kong.

**Formal analysis:** Ling Kong.

**Funding acquisition:** Ling Kong.

**Methodology:** Hua Zou.

**Project administration:** XiaoHui Xu.

**Resources:** XiaoHui Xu.

**Supervision:** XiaoHui Xu.

**Validation:** WenYuan Xu.

**Writing – original draft:** Ling Kong.

**Writing – review & editing:** Hua Zou, WenYuan Xu, XiaoHui Xu.






