# Supplementary material for: The causal effect of juvenile idiopathic arthritis on IgA nephropathy: A Mendelian randomization study
Source: Medicine (Baltimore). 2026 Jun 26;105(26):e48981. doi: 10.1097/MD.0000000000048981 (PMC13313782; doi:10.1097/MD.0000000000048981)
Supplement: Supplementary file 1 [file medi-105-e48981-s001.docx]

Supplementary Table 1. Index instrumental SNPs for juvenile idiopathic arthritis (JIA, primary data set) and the effects, standard errors on IgA nephropathy (IgAN) in the discovery analysis.

| SNP | chr | Effect  allele | Other  allele | beta.JIA | se.JIA | pval.JIA | F-statistics | beta.IgAN | se.IgAN | pval.IgAN | Ncase  IgAN | Ncontrol  IgAN | Ncase  JIA | ncontrol.  JIA |
| --- | --- | --- | --- | --- | --- | --- | --- | --- | --- | --- | --- | --- | --- | --- |
| rs112104961 | 6 | G | T | -0.2927 | 0.045933 | 1.86E-10 | 40.60142 | -0.1809 | 0.0474 | 0.000135 | 5556 | 21178 | 3305 | 9196 |
| rs113274764 | 6 | T | C | 0.4830 | 0.054961 | 1.52E-18 | 77.21885 | 0.0699 | 0.071 | 0.3252 | 5556 | 21178 | 3305 | 9196 |
| rs114121446 | 6 | G | A | -0.7230 | 0.104095 | 3.76E-12 | 48.23908 | 0.2093 | 0.1379 | 0.1291 | 5556 | 21178 | 3305 | 9196 |
| rs114933571 | 6 | G | A | 1.1312 | 0.043245 | 7.90E-151 | 684.1541 | -0.1475 | 0.1515 | 0.3302 | 5556 | 21178 | 3305 | 9196 |
| rs116234817 | 6 | A | G | 0.7390 | 0.050696 | 3.94E-48 | 212.4513 | -0.2961 | 0.3408 | 0.3849 | 5556 | 21178 | 3305 | 9196 |
| rs11889341 | 2 | T | C | 0.2128 | 0.033378 | 1.83E-10 | 40.6346 | 0.0102 | 0.029 | 0.7265 | 5556 | 21178 | 3305 | 9196 |
| rs12110785 | 6 | C | T | 0.4481 | 0.038511 | 2.75E-31 | 135.3406 | 0.0652 | 0.0403 | 0.1058 | 5556 | 21178 | 3305 | 9196 |
| rs2517828 | 6 | G | A | 0.3279 | 0.028049 | 1.42E-31 | 136.6578 | -0.0976 | 0.0655 | 0.1361 | 5556 | 21178 | 3305 | 9196 |
| rs2856680 | 6 | G | C | -0.6029 | 0.055946 | 4.46E-27 | 116.1095 | -0.3072 | 0.06 | 3.07E-07 | 5556 | 21178 | 3305 | 9196 |
| rs3131059 | 6 | C | T | 0.1544 | 0.028023 | 3.60E-08 | 30.34986 | -0.0086 | 0.0334 | 0.7963 | 5556 | 21178 | 3305 | 9196 |
| rs3134963 | 6 | C | T | 0.4135 | 0.04448 | 1.44E-20 | 86.42264 | 0.1535 | 0.0542 | 0.004577 | 5556 | 21178 | 3305 | 9196 |
| rs34536443 | 19 | C | G | -0.4275 | 0.071564 | 2.32E-09 | 35.68128 | -0.1316 | 0.084 | 0.1172 | 5556 | 21178 | 3305 | 9196 |
| rs6679677 | 1 | A | C | 0.3042 | 0.040817 | 9.18E-14 | 55.52526 | 0.0534 | 0.044 | 0.2254 | 5556 | 21178 | 3305 | 9196 |
| rs74379225 | 6 | G | A | 0.3067 | 0.042172 | 3.50E-13 | 52.89689 | 0.0771 | 0.1049 | 0.4625 | 5556 | 21178 | 3305 | 9196 |
| rs7731626 | 5 | A | G | -0.1991 | 0.027029 | 1.76E-13 | 54.24941 | -0.1245 | 0.0516 | 0.01586 | 5556 | 21178 | 3305 | 9196 |
| rs8192591 | 6 | T | C | -0.5432 | 0.087766 | 6.04E-10 | 38.30132 | -0.2697 | 0.0799 | 0.000738 | 5556 | 21178 | 3305 | 9196 |
| rs9277625 | 6 | T | C | 0.5138 | 0.039984 | 8.65E-38 | 165.0859 | 0.0398 | 0.0442 | 0.3683 | 5556 | 21178 | 3305 | 9196 |
| rs9461778 | 6 | G | A | 0.5713 | 0.034264 | 2.04E-62 | 277.9678 | 0.1616 | 0.0678 | 0.01715 | 5556 | 21178 | 3305 | 9196 |
| rs9960807 | 18 | G | A | 0.2298 | 0.038077 | 1.58E-09 | 36.4332 | 0.0496 | 0.038 | 0.1925 | 5556 | 21178 | 3305 | 9196 |

SNP, single nucleotide polymorphism; chr, chromosome; se, standard error; IgAN, IgA nephropathy, JIA, juvenile idiopathic arthritis. IgAN GWAS data was from Kiryluk K et al.^1^, and JIA GWAS data from López-Isac E et.al. ^2^

1. Kiryluk K, Sanchez-Rodriguez E, Zhou XJ, et al. Genome-wide association analyses define pathogenic signaling pathways and prioritize drug targets for IgA nephropathy. *Nat Genet*. Jul 2023;55(7):1091–1105. doi:10.1038/s41588-023-01422-x

2. Lopez-Isac E, Smith SL, Marion MC, et al. Combined genetic analysis of juvenile idiopathic arthritis clinical subtypes identifies novel risk loci, target genes and key regulatory mechanisms. *Ann Rheum Dis*. Mar 2021;80(3):321–328. doi:10.1136/annrheumdis-2020-218481
