# Supplementary material for: The causal effect of juvenile idiopathic arthritis on IgA nephropathy: A Mendelian randomization study
Source: Medicine (Baltimore). 2026 Jun 26;105(26):e48981. doi: 10.1097/MD.0000000000048981 (PMC13313782; doi:10.1097/MD.0000000000048981)
Supplement: Supplementary file 2 [file medi-105-e48981-s002.docx]

Supplementary Table 2. Index instrumental SNPs for IgA nephropathy (IgAN) and the effects, standard errors on juvenile idiopathic arthritis (JIA, primary data set) in the reverse MR analysis.

| SNP | chr | effect_allele | other_allele | beta.IgAN | se.IgAN | pval_IgAN | F-statistics | beta.JIA | pval.JIA | se.JIA |
| --- | --- | --- | --- | --- | --- | --- | --- | --- | --- | --- |
| rs10065637 | 5 | T | C | -0.1737 | 0.0323 | 7.78E-08 | 28.91553 | -0.18707 | 1.12E-08 | 0.03275 |
| rs117076176 | 8 | T | C | 0.5319 | 0.1063 | 5.67E-07 | 25.03399 | 0.03974 | 0.640545 | 0.085107 |
| rs1466226 | 8 | T | C | 0.1598 | 0.0314 | 3.70E-07 | 25.89589 | 0.057352 | 0.053137 | 0.029657 |
| rs2040762 | 7 | T | C | 0.2419 | 0.0483 | 5.37E-07 | 25.07922 | -0.03704 | 0.246832 | 0.031984 |
| rs3128927 | 6 | T | C | -0.2513 | 0.0286 | 1.73E-18 | 77.19505 | -0.08455 | 0.024259 | 0.037529 |
| rs4077515 | 9 | T | C | 0.1551 | 0.0245 | 2.46E-10 | 40.0708 | -0.01618 | 0.682303 | 0.039534 |
| rs4273077 | 17 | A | G | -0.2265 | 0.0454 | 5.93E-07 | 24.88635 | -0.03864 | 0.504966 | 0.057963 |
| rs4648011 | 4 | T | G | 0.1271 | 0.0255 | 6.27E-07 | 24.83975 | 0.013005 | 0.641186 | 0.027905 |
| rs58905141 | 6 | A | G | -0.377 | 0.0757 | 6.43E-07 | 24.79862 | -0.08692 | 0.270357 | 0.078858 |
| rs6677604 | 1 | A | G | -0.2167 | 0.0313 | 4.43E-12 | 47.92539 | 0.001499 | 0.871698 | 0.009281 |
| rs67898294 | 16 | T | C | -0.2169 | 0.0344 | 3.04E-10 | 39.75015 | 0.048762 | 0.327334 | 0.049782 |
| rs75152619 | 18 | T | C | -0.4877 | 0.0996 | 9.88E-07 | 23.97305 | -0.03075 | 0.565514 | 0.053513 |
| rs7525284 | 1 | A | G | 0.1474 | 0.0277 | 1.05E-07 | 28.31209 | 1.00E-05 | 0.84948 | 5.27E-05 |
| rs9268557 | 6 | T | C | -0.3166 | 0.0245 | 4.25E-38 | 166.9653 | -0.1342 | 5.94E-07 | 0.026877 |

SNP, single nucleotide polymorphism; chr, chromosome; se, standard error; IgAN, IgA nephropathy, JIA, juvenile idiopathic arthritis. IgAN GWAS data was from Kiryluk K et al.^1^, and JIA GWAS data from López-Isac E et.al. ^2^

1. Kiryluk K, Sanchez-Rodriguez E, Zhou XJ, et al. Genome-wide association analyses define pathogenic signaling pathways and prioritize drug targets for IgA nephropathy. *Nat Genet*. Jul 2023;55(7):1091–1105. doi:10.1038/s41588-023-01422-x

2. Lopez-Isac E, Smith SL, Marion MC, et al. Combined genetic analysis of juvenile idiopathic arthritis clinical subtypes identifies novel risk loci, target genes and key regulatory mechanisms. *Ann Rheum Dis*. Mar 2021;80(3):321–328. doi:10.1136/annrheumdis-2020-218481
