# Supplementary material for: The causal effect of juvenile idiopathic arthritis on IgA nephropathy: A Mendelian randomization study
Source: Medicine (Baltimore). 2026 Jun 26;105(26):e48981. doi: 10.1097/MD.0000000000048981 (PMC13313782; doi:10.1097/MD.0000000000048981)
Supplement: Supplementary file 3 [file medi-105-e48981-s003.docx]

Supplementary Table 3 Index instrumental SNPs for juvenile idiopathic arthritis (JIA, validation data set) and the effects, standard errors on IgA nephropathy (IgAN) in the reverse MR analysis.

| SNP | chr | effect_allele | other_allele | beta.IgAN | se.IgAN | pval.IgAN | F-statistics | beta.JIA | se.JIA | pval.JIA |
| --- | --- | --- | --- | --- | --- | --- | --- | --- | --- | --- |
| rs10065637 | 5 | T | C | -0.1737 | 0.0323 | 7.78E-08 | 28.91553 | -0.31924 | 0.072255 | 9.95E-06 |
| rs117076176 | 8 | T | C | 0.5319 | 0.1063 | 5.67E-07 | 25.03399 | 0.232698 | 0.178315 | 0.1919 |
| rs3128927 | 6 | T | C | -0.2513 | 0.0286 | 1.73E-18 | 77.19505 | -0.05964 | 0.060976 | 0.328 |
| rs4077515 | 9 | T | C | 0.1551 | 0.0245 | 2.46E-10 | 40.0708 | 0.018822 | 0.053974 | 0.727301 |
| rs4648011 | 4 | T | G | 0.1271 | 0.0255 | 6.27E-07 | 24.83975 | -0.02469 | 0.053289 | 0.643101 |
| rs58905141* | 6 | A | G | -0.377 | 0.0757 | 6.43E-07 | 24.79862 | -0.27308 | 0.13802 | 0.04787 |
| rs6677604 | 1 | A | G | -0.2167 | 0.0313 | 4.43E-12 | 47.92539 | 0.042101 | 0.066926 | 0.5293 |
| rs67898294 | 16 | T | C | -0.2169 | 0.0344 | 3.04E-10 | 39.75015 | -0.00702 | 0.075224 | 0.9256 |
| rs7525284 | 1 | A | G | 0.1474 | 0.0277 | 1.05E-07 | 28.31209 | -0.01228 | 0.060838 | 0.8401 |
| rs9268557 | 6 | T | C | -0.3166 | 0.0245 | 4.25E-38 | 166.9653 | -0.07881 | 0.053793 | 0.1429 |

The outcome effects of SNP rs58905141 was proxied by SNP rs79411652. SNP, single nucleotide polymorphism; chr, chromosome; se, standard error; IgAN, IgA nephropathy, JIA, juvenile idiopathic arthritis. IgAN GWAS data was from Kiryluk K et al.^1^, and JIA GWAS data from Hinks A et al. ^2^

1. Kiryluk K, Sanchez-Rodriguez E, Zhou XJ, et al. Genome-wide association analyses define pathogenic signaling pathways and prioritize drug targets for IgA nephropathy. *Nat Genet*. Jul 2023;55(7):1091–1105. doi:10.1038/s41588-023-01422-x

2. Hinks A, Cobb J, Marion MC, et al. Dense genotyping of immune-related disease regions identifies 14 new susceptibility loci for juvenile idiopathic arthritis. *Nat Genet*. Jun 2013;45(6):664–9. doi:10.1038/ng.2614
